# Chronoamperometric study of elemental sulphur (S) nanoparticles (NPs) in NaCl water solution: new methodology for S NPs sizing and detection

**DOI:** 10.1186/s12932-015-0016-2

**Published:** 2015-02-12

**Authors:** Elvira Bura-Nakić, Marija Marguš, Darija Jurašin, Ivana Milanović, Irena Ciglenečki-Jušić

**Affiliations:** Center for Marine and Environmental Research, Ruđer Bošković Institute, Bijenička 54, 10 000 Zagreb, Croatia; Division of Physical Chemistry, Ruđer Bošković Institute, Bijenička 54, 10 000 Zagreb, Croatia

## Abstract

**Background:**

Elemental sulfur (S) persists in natural aquatic environment in a variety of forms with different size distributions from dissolved to particulate. Determination of S speciation mainly consists of the application of chromatographic and electrochemical techniques while its size determination is limited only to the application of microscopic and light scattering techniques. S biological and geochemical importance together with recent increases of S industrial applications requires the development of different analytical tools for S sizing and quantification. In recent years the use of electrochemical measurements as a direct, fast, and inexpensive technique for the different nanoparticles (NPs) characterization (Ag, Au, Pt) is increasing. In this work, electrochemical i.e. chronoamperometric measurements at the Hg electrode are performed for determination of the size distribution of the S NPs.

**Results:**

S NPs were synthesized in aqueous medium by sodium polysulphide acidic hydrolysis. Chronoamperometric measurements reveal the polydisperse nature of the formed suspension of S NPs. The electrochemical results were compared with dynamic light scattering measurements parallel run in the same S NPs suspensions. The two methods show fairly good agreement, both suggesting a log-normal size distribution of the S NPs sizes characterized by similar parameters.

**Conclusions:**

The preliminary results highlight the amperometric measurements as a promising tool for the size determination of the S NPs in the water environment.

## Introduction

Elemental sulphur (S in further text) is an important element, having many practical applications when present as NPs. Examples of its application are fungicides in agriculture or in agrochemical industry [1-4], nanocomposites of Li-ion batteries, S nanowires in hybrid materials, production of plastics or sulphuric acid, and in the pharmaceutical industry [5-10].

As with other NPs the size of S NPs has an important role effecting their properties and utilizations. Recent studies show that the application of nanoparticulate S as a fungicide is more effective than the use of micron-sized S, due to the increased surface/volume ratio and enhanced surface energy density of the NPs [3,4].

The synthesis of S NPs can be carried out by various methods in different media: in microemulsions from sublimed sulfur, and in an aqueous medium with the use of surfactants or electrochemical synthesis [11-22]. Colloidal or nano-sized sulphur particles can be prepared in different ways such as the acidic hydrolysis of sodium thiosulphate, solvent/non-solvent precipitation method, the acidic decomposition of the polysulphides, the reduction of H_2_S by Fe-chelate, or the synthesis of S-cysteine colloidal solutions by ultrasonic treatment [11-22]. The effects of different experimental conditions such as reactant concentration, temperature, sonication, types of used surfactants, and their concentration are found to influence growth kinetics of S NPs [19-22]. The coarsening rate constant was found to be highly dependent on the type of acid used as a catalyst [21,22].

In available studies, the size distribution of the synthesized S NPs were characterized mainly by the use of so called “state-of-the-art techniques”, i.e. atomic force microscopy (AFM), high-resolution transmission electron microscopy (HR-TEM), Fourier transform infrared (FT-IR) spectroscopy, energy dispersive X-ray (EDX) spectroscopy, or environmental scanning electron microscopy [11-16,19-22]. However, nowdays due to increased S NPs production and its increasing application as “eco-safe” antifungal agents, an urgent need has emerged for developing a more cost efficient, easy, and quick methodology for the S NPs characterization and determination in the water environment.

At the same time, elemental S (particulate, colloidal, and dissolved) is recognized as a very important S species in biogeochemical S cycling in aquatic environment. There it can be produced by microbial activity as well as by chemical reactions catalyzed by mineral phases MnO_2_/Fe_2_O_3_ [23,24]. So far, most studies on elemental S have been carried out in sediments and very few in the water column [25-31]. In these studies different methods and experimental approaches for elemental S determination were employed, mostly electrochemistry and HPLC with UV/VIS detection [25-31]. However it appears as though none of the used methods can directly, without sample pretreatment differentiate between colloidal, dissolved and particulate elemental S.

Therefore, the main goal of this study is to investigate possibilities for using electrochemistry as a tool for the fast and direct (without sample pretreatment) quantification and sizing of S NPs in the water environment. Based on the previously performed chronoamperometric measurements of FeS and PbS NPs at the Hg electrode in sodium chloride solutions [32,33], a similar methodology is suggested for S NPs determination.

Elemental S NPs were prepared directly in the electrochemical cell by the acidic decomposition of sodium tetrasulphide. In available literature it’s well established that acidification of polysulfane, thiosulfate as well as polysulfide solution will produce elemental S NPs [3,12,14,19,20]. Due to low solubility of elemental S in water, acid decomposition of S_x_^2−^ will cause precipitation of elemental S in a colloidal form. In the present study we didn’t investigated shape of the formed elemental S NPs however literature data and microscopic images indicate that elemental SNPs produced by acid decomposition of different sulfur species are spherical in shape [3,12,19,20].

The size of the produced S NPs, were monitored by dynamic light scattering (DLS) measurements. The DLS method is widely used as an effective technique to determine size of NPs in suspensions. Also, DLS is chosen because enables determination of size distribution during aging time in parallel with electrochemical measurements without affecting the sample.

## Materials and methods

The suspensions of S NPs for both the electrochemical and the DLS measurements were prepared directly in the electrochemical cell by acidification (to *p*H ≈ 2 by HCl, Kemika, Croatia) of sodium tetrasulphide (Na_2_S_4_) (Alfa Aesar, USA) solutions in deaerated 0.55 mol∙dm^−3^ NaCl (Kemika, Croatia) used as supporting electrolyte. All electrochemical and DLS measurements were performed 10 min after acidification of polysulfide. The polysulphide stock solutions were prepared by dissolving crystals of Na_2_S_4_ in Milli-Q water (*p*H ≈ 10, adjusted by NaOH (Kemika, Croatia) previously deaerated by N_2_. All the chemicals used were of reagent grade.

The electrochemical measurements were performed with an Autolab PGSTAT128N potentiostat (Eco Chemie, Utrecht, Netherlands) in combination with a multimode electrode Stand VA 663 (Metrohm, Herisau, Switzerland). The Hg electrode was used in the SMDE mode in all our measurements. A Pt rod served as an auxiliary electrode and Ag/AgCl (in 3 mol∙dm^−3^ KCl solution, Kemika, Croatia) was applied as a reference electrode. The volume of the electrochemical cell was 50 cm^3^.

Chronoamperometric measurements, where a step potential is applied and the current (*i)* is measured as a function of time (*t)* at a fixed potential between the working and reference electrode were performed on a single Hg drop at following instrumental parameters: 1) applied potential of −0.8 V (vs. Ag/AgCl) was selected due to recorded highest frequency of impact events at that potential; 2) sampling or interval time was 0,1 s; 3) measurement duration was 60 s; 4) equilibration time was 1 s; and 5) current range during measurement was 100 nA – 1 μA.

Observed *i-t* response is usually combination of two components: capacitative current related to the charging the double-layer and Faradaic current related to the electron transfer reaction e.g. sharp current transients due to Faradaic charge transfer while the NPs is in a contact with Hg electrode. In our case collision of S NPs with Hg electrode followed by the reduction of the colliding S NPs cause appearance of the transient current signals superimposed on the chronoamperometric *i/t* curve [32,33].

All chronoamperometric curves were analyzed in the same way to eliminate possible influence of noise; the baseline was removed and the transient current peaks (e.g. spikes) with height exceeding a threshold of 0.3 nA were integrated.

In voltammetric measurements, current is measured while scanning the entire voltage range of the electrode, i.e. (*i* – *E*) response, in our case from −1000 to −400 mV, which allows the measurements of more than one species at a given time in the same region of space [27,30,31].

The S NPs size distribution was measured using Zetasizer Nano ZS (Malvern, UK) equipped with green laser (532 nm). Intensity of scattered light was detected at the angle of 173°. The mean hydrodynamic radius, from now on referred as mean radius (*r*), was estimated using the *Stokes–Einstein* equation, *D* = *k*_B_*T*/6π*ηr* (where *k*_B_ is the *Boltzmann* constant, *T* is the temperature, *η* is the viscosity of the dispersing medium, and *D* is the apparent diffusion coefficient) under the assumption that the particle exists as a compact sphere. Mean radii, always based on six or more measurements with a relative standard deviation of ±15%, were derived from distribution by volume. All measurements were performed at 20–25 °C.

## Results and discussion

### Voltammetric and chronoamperometric study of the polysulphide solution at the Hg electrode

**Figure 1:**
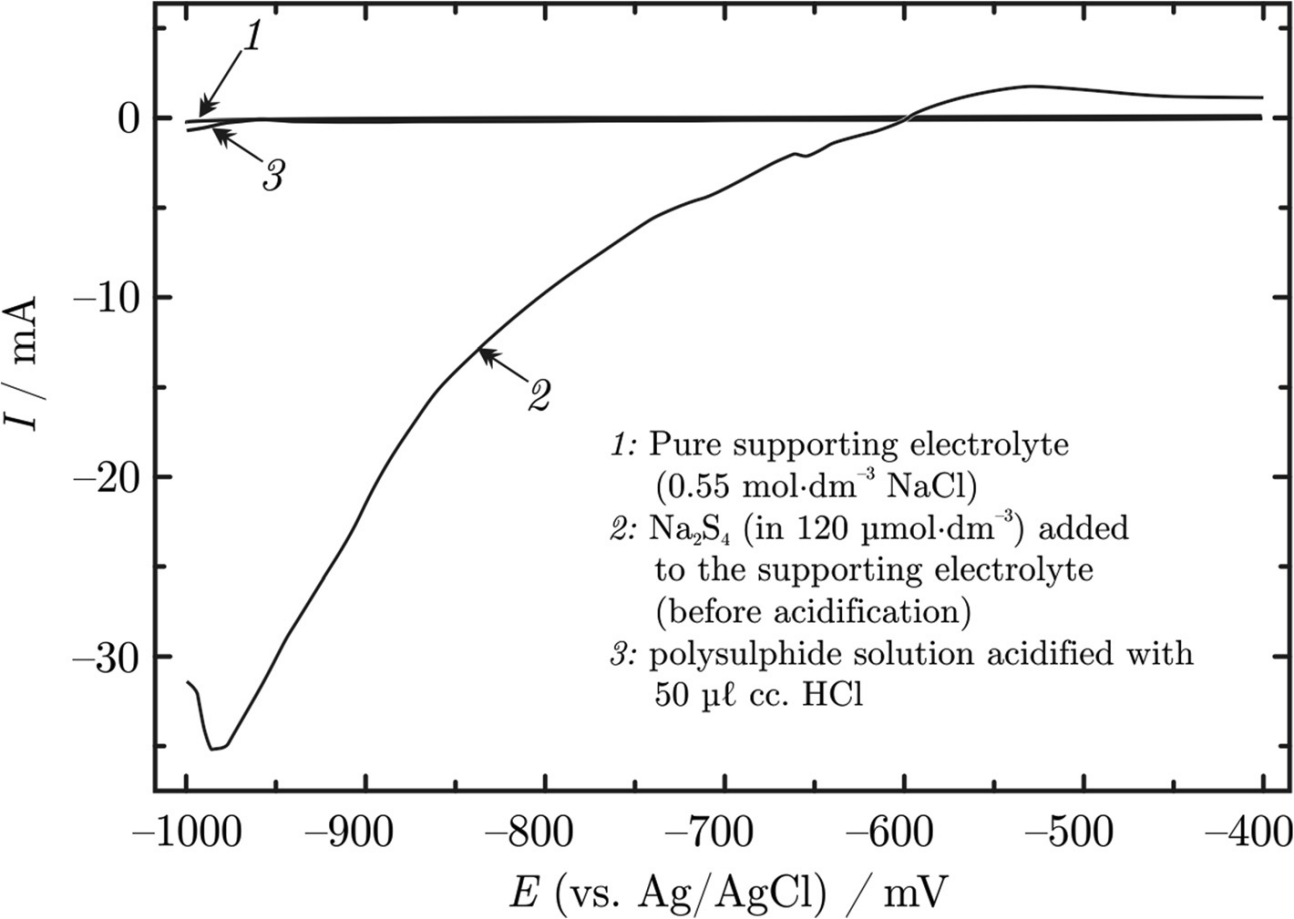
**Sampled DC voltammograms recorded at the Hg in the solution of pure supporting electrolyte (curve 1), and in the Na**_**2**_ **S**_**4**_ **solution before (curve 2) and after (curve 3) acidification.** The voltammograms are recorded between −1000 mV and −400 mV vs. Ag/AgCl with potential steps of 5.1 mV.

Figure 1 shows sampled DC voltammograms measured in: 1) pure 0.55 mol∙dm^−3^ NaCl supporting electrolyte (curve 1); 2) the supporting electrolyte containing tetrasulphide in concentration of 120 μmol∙dm^−3^, at *p*H ≈ 8 (curve 2); and 3) acidified tetrasulphide solution of same concentration, at pH ≈ 2 (curve 3). The measurements were carried out in the same manner in all the three cases, i.e. the potential was changed stepwise (in steps of 5.1 mV) from a starting potential of −1000 mV (vs. Ag/AgCl) to the positive direction until the end potential of −400 mV was reached.

The voltammogram recorded in the pure 0.55 mol∙dm^−3^ NaCl solution (curve 1 in Figure 1) shows no observable features through the entire studied potential range. Contrarily, the voltammogram measured in the polysulphide containing solution (curve 2) is characterized by the appearance of a cathodic current in the potential range from −1000 mV to −600 mV, while at potentials more positive than −600 mV, an anodic current (reaching a plateau near −580 mV) is revealed. The appearance of the cathodic current in the negative potential range implies the reduction process according to the equation (1) [34,35]:

1 $$ {S}_{\mathrm{n}}^{2-}+2\ \left(\mathrm{n}-1\right)\ {e}^{-}+\mathrm{n}\ {H}_2O\to \mathrm{n}H{S}^{-}+\mathrm{n}O{H}^{-} $$

The revealed anodic current at potentials more positive than −600 mV can be assigned to the well known oxidation of the Hg by sulphide according to equation (2) [33-36]:

2 $$ H{S}^{-}+Hg\to HgS+2{e}^{-}+{H}^{+} $$

In the polysulphide solution, in the potential range −1000 mV < *E* < −900 mV, an appearance of a local (cathodic) current maximum may also be revealed. This feature was reported earlier and was assigned to the adsorption of an unidentified sulphur species [34].

By acidifying the polysulphidic solution at *p*H ≈ 2, the cathodic and anodic currents disappeared (curve 3 in Figure 1), due to polysulphide disproportionation to sulphide and elemental sulphur according to equilibrium reaction (3) [30,34-39]:

3 $$ {S}_{\mathrm{x}+1}^{2-}+{\mathrm{H}}^{+}\leftrightarrows {\scriptscriptstyle \frac{\mathrm{x}}{8}}{\mathrm{S}}_8+\mathrm{H}{\mathrm{S}}^{-} $$

**Figure 2:**
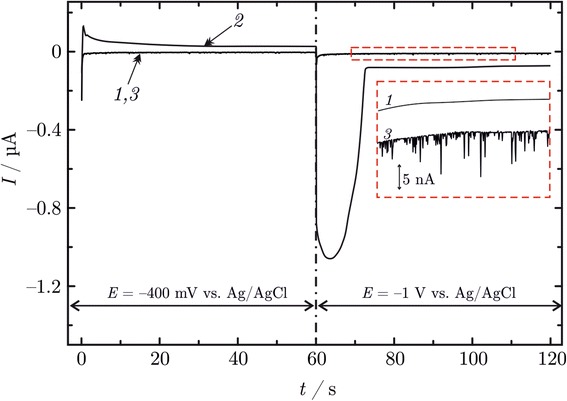
**Chronomperometric curves recorded in a solution of pure supporting electrolyte (curve 1), and in a Na**_**2**_ **S**_**4**_ **solution before (curve 2) and after (curve 3) acidification.** After 60 s, the electrode potential was changed from −400 mV to −1000 mV as indicated. A given region of the curves 1 and 3 is shown magnified in the inset with visible spikes arising from S NPs impacts.

In Figure 2, the chronoamperometric measurements for the same three polysulfide systems are presented: (curve 1) the pure supporting electrolyte, (curve 2) the electrolyte containing polysulphides before (curve 2), and (curve 3) after acidification. These measurements were performed in such way that the electrode potential was set to −400 mV vs. Ag/AgCl, and the current was monitored for 60 s. Thereafter, the potential was switched to −1000 mV and the current was continuously monitored for another 60 s.

Similarly as recorded in sampled DC voltammograms, the chronoamperometric curves were relatively featureless when measured in the pure supporting electrolyte (curve 1). However, in the electrolyte containing polysulphide (curve 2), again the anodic current was measured when the electrode potential was set to −400 mV. This current is most probably maintained by the formation of HgS according to equation (2). Switching the potential from −400 mV to −1000 mV caused a large cathodic current which is undoubtedly caused by the reduction of the previously formed HgS. After HgS is completely reduced, a cathodic current of relatively small and constant value was recorded. This current is controlled by the diffusion of polysulphide ions from the bulk electrolyte to the Hg electrode surface, followed by reduction of S(0) from polysulfide molecule according to equation (1).

After acidification, both the cathodic currents measured at −1000 mV and the anodic currents measured at −400 mV disappeared, and the recorded curve (curve 3) resembles that obtained from the pure NaCl supporting electrolyte (curve 1), similarly to previously observed with the sampled DC voltammetry. However, a close look at recorded curves enlarged in the inset of Figure 2 reveal the sharp current transients in the case of the acidified polysulphide solution. These spikes are assigned to the reduction of elemental S NPs including aggregates that were formed during the acidification of the polysulphide solution in reaction (3) [30,34-38]. Spikes were not visible in the curves recorded in the pure supporting electrolyte.

The above experiment was repeated at different potential values, and it was found that in acidic media (*p*H ≈ 2), sharp reduction current transients can be recorded in the whole investigated potential range −300 mV < *E* < −1500 mV. At higher *p*H around 8, the potential window with recorded sharp transients was shifted towards more negative values in the potential range −800 mV < *E* < −1900 mV. Such results imply that H^+^ ions are involved in the reduction of S NPs according to the reaction (4) which is responsible for the recorded cathodic transients:

4 $$ {S}^0+{H}^{+}+2{e}^{-}\to H{S}^{-} $$

The observed phenomenon in relation with results obtained with FeS NPs [32] offers an opportunity for further study on possible S NPs sizing by chronoamperometric measurements.

### Determination of S NPs size distribution by dynamic light scattering (DLS)

**Figure 3:**
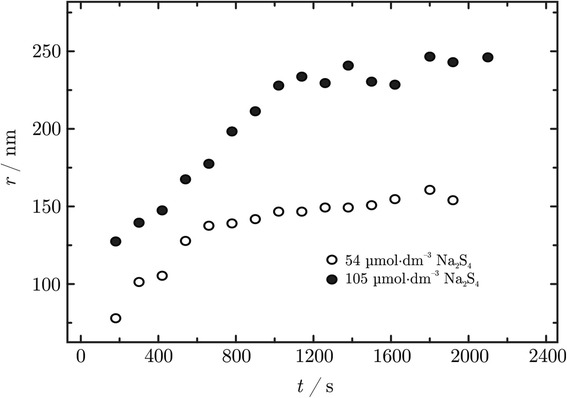
**Variation of S NPs mean radii (** ***r*** **) with ageing time (** ***t*** **) determined by DLS measurements.** The S NPs were prepared by the acidification of the Na_2_S_4_ solutions with HCl to *p*H ≈ 2. During the first 30 min of ageing, a significant coarsening of the particles can be observed.

The suspensions of S NPs for DLS measurements were prepared by acidifying 54 and 105 μmol∙dm^−3^ Na_2_S_4_ in 0.55 mol∙dm^−3^ NaCl solutions. When measured directly after acidification, the mean radius of the formed S NPs in the two solutions was found to be ~50 nm and ~110 nm, respectively. In both suspensions the formed S NPs grow with ageing. With time their size exceeds 150 and 250 nm, respectively (Figure 3) indicating possible formation of aggregates. The observed coarsening was also influenced by higher concentration of the NaCl solution that diminishes ζ-potentials and the inter-particle electrostatic repulsion, and facilitates aggregation during aging as already observed with PbS, HgS, Cu xS and FeS NPs [32,33,40-42].

**Figure 4:**
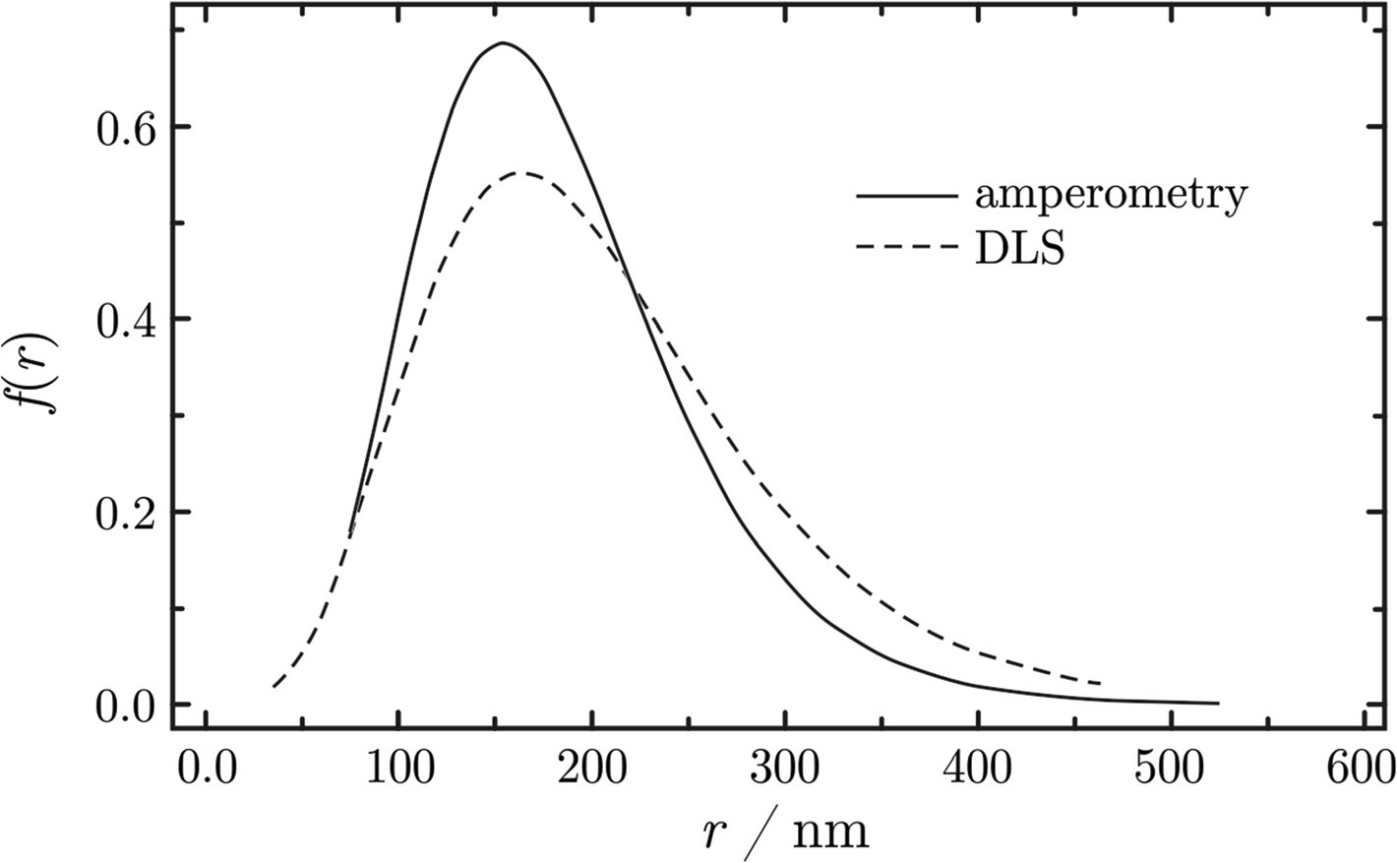
**The log-normal probability distribution functions (eq.** **7** **) obtained from the results of DLS and chronoamperometric measurements.** The values of the used parameters are listed in Table 1.

It is important to mention that volume-weighted size distribution of the formed S NPs indicate polydispersity of the investigated suspensions (see later Figure 4). This fact has to be considered in the interpretation of the all electrochemical results later in the Section 3.3.

### Determintion of S NPs size distribution by chronoamperometry

**Figure 5:**
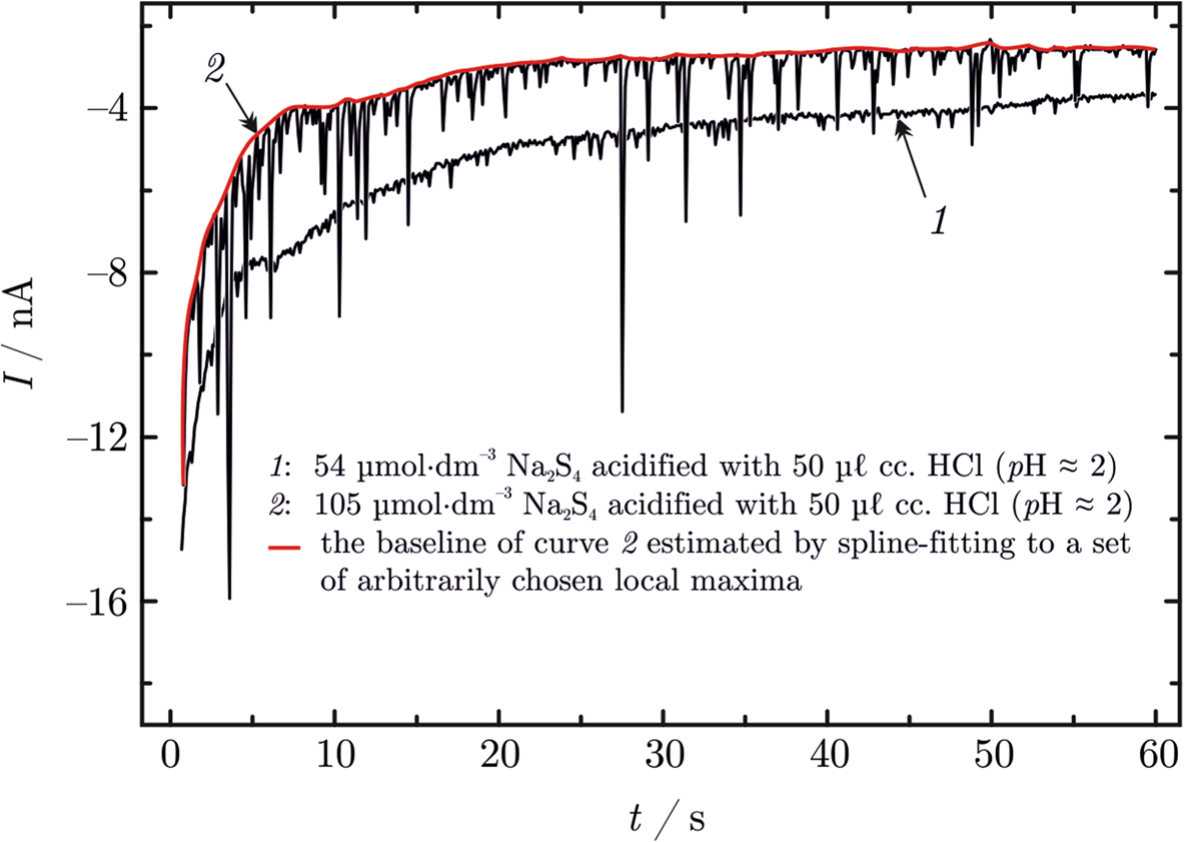
**Chronoamperometric curves recorded in Na**_**2**_ **S**_**4**_ **0.55 mol∙dm** ^**−3**^ **NaCl solutions of different concentrations 10 minutes after acidification with HCl.** The potential of the Hg electrode was set to −800 mV vs. Ag/AgCl.

The current vs. time curves recorded in acidified solutions containing 54 μmol∙dm^−3^ (curve 1) and 105 μmol∙dm^−3^ (curve 2) Na_2_S_4,_ previously measured by DLS are presented in Figure 5. It is clear that in the more diluted solution, the reduction current transients have much smaller charges, implying formation of the smaller S NPs in accordance with the DLS measurements presented in Figure 3. In order to estimate the size of the formed S NPs the charge (*Q)* corresponding to the impact events were estimated in a way that the baseline from the recorded chronoamperogram from Figure 5 (curve 2) was removed, and the transient current peaks with height exceeding a threshold of 0.3 nA measured from the baseline were integrated. Assuming that the S NPs are spherical with radius r, the maximum charge passed as a result of the complete S NPs reduction, according to reaction (4), can be given by the Equation (5):

5 $$ \mathrm{r}=\sqrt[3]{\frac{3{\mathrm{M}}_{\mathrm{S}}\mathrm{Q}}{8\mathrm{F}\pi \rho }} $$

where M_S_ = 32 *g* ⋅ *mol*^− 1^ is the molar mass, *ρ* ≈ 2 *g* ⋅ *cm*^− 3^ is the approximate bulk density of the S, radius, *r* of the colliding S NPs can be determined from the recorded charge, *Q* yielded during S NPs reduction [43].

**Figure 6:**
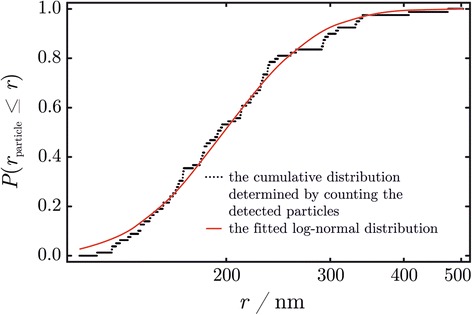
**The discrete cumulative distribution of the determined particle radii and the lognormal distribution fitted to it.** The parameters of the fitted curve are presented in Table 1.

Figure 6 shows a (discrete) cumulative distribution of the calculated particle radii from Equation 5 that was obtained by calculating the probability of those particles radii that are smaller or equal to a given value of r. The discrete cumulative distribution presented in Figure 6 can be described reasonably well with a log-normal distribution of the form

6 $$ \mathrm{P}\left({\mathrm{r}}_{particle}\le \mathrm{r}\right)={\displaystyle \underset{0}{\overset{r}{\int }}\mathrm{f}\left(\mathrm{x}\right)}\mathrm{d}\mathrm{x}, $$

where

7 $$ \mathrm{f}\left(\mathrm{x}\right)=\frac{1}{\mathrm{x}\sqrt{2\pi {\sigma}^2}}{\mathrm{e}}^{-\frac{{\left( \ln {\scriptscriptstyle \raisebox{1ex}{$\mathrm{x}$}\!\left/ \!\raisebox{-1ex}{$\mathrm{m}$}\right.}-\mu \right)}^2}{2{\sigma}^2}}. $$

with parameters *μ* (mean) an *σ* (standard deviation). The function (6) was fitted to the points of the discrete cumulative distribution by the use of the Levenberg–Marquardt method (see the red curve in Figure 6) [44].

The same treatment was applied to particle size data obtained from DLS measurements run in parallel in the same S NPs solution. For this case the parameters *μ* and *σ* were also determined, and values were found to be in a fairly good agreement with those determined by the chronoamperometric reduction transients detection. Table 1 shows all distribution parameters from plots in Figure 4 illustrating the probability distribution calculated by equation 7 as well as the DLS and the chronoamperometric measurements.

**Table 1:** **Parameters obtained by fitting the cumulative distribution of particle sizes determined by the DLS and amperometric measurements with a lognormal cumulative distribution function of the form (** 6 **)**

**Parameters of the lognormal distribution**	**Amperometry**	**DLS**
Median (e^*μ*^ / μm)	0.208 ± 0.019	0.2250 ± 0.0029
Shape (σ^2^)	0.291 ± 0.065	0.341 ± 0.0091
Mean radius $\left({\mathrm{e}}^{\mu +{\sigma}^2/2}\right)$	0.2406 ± 0.0091	0.2667 ± 0.0014

Confidence level: 95%.

### Potential for future application of chronoamperometric measurements in sizing and detection of S in natural waters

Based on the already published data on chronoamperometric study of FeS NPs [32] and results from this study showing that S NPs size determined by the chronoamperometric measurements are in the fairly good agreement with the parallel run DLS, it can be stated that electrochemistry, in comparison with other more expensive and sophisticated methods, is a promising alternative for the direct size determination of the S NPs in water solutions.

Due to high affinity of Hg towards sulfur compounds, the application of chronoamperometric measurement for S NPs characterization in the natural water environment is very promising. However, we are aware of some difficulties related to natural environmental conditions which could influence the behavior and fate of S NPs. Amongst, are the most important interaction with the natural organic matter (NOM), fast aggregation and settling of the S NPs due to relatively high ionic strength conditions. Possible interferences which can rise from presence of other metal sulfide NPs like FeS can be avoided by careful choice of the experimental conditions, i.e. applied potential. We already showed that FeS and PbS NPs will produce spike like signals only in the narrow potential range allowing their distinction from the colloidal S NPs [32,33].

The chosen expression of the measured data in the form of cumulative and log-normal probability distribution functions may also lead to a better interpretation of the obtained results as compared to the more standard way of presenting the size distributions in the form of histograms [32,43,45-47]. Although histograms reveal the range over which the particle sizes are distributed, often due to the relatively arbitrary selection of bins, histograms are insufficient for finding a mathematical formula describing the size distribution. On the contrary, the current data presentation offers new perspectives for NPs analysis by use of the model distributions.

The S NPs electrochemical behavior at different *p*H together with the applicability of the method in relation to the size detection limit and study in real relevant environmental samples are planned as a next step in the further investigations.
